# Quantitative MRI Assessment of Post-Surgical Spinal Cord Injury Through Radiomic Analysis

**DOI:** 10.3390/jimaging10120312

**Published:** 2024-12-08

**Authors:** Azadeh Sharafi, Andrew P. Klein, Kevin M. Koch

**Affiliations:** Radiology Department, Medical College of Wisconsin, Milwaukee, WI 53226, USA; aklein@mcw.edu (A.P.K.); kmkoch@mcw.edu (K.M.K.)

**Keywords:** radiomics, spinal cord injury, multi-spectral imaging, magnetic resonance imaging, metal artifact

## Abstract

This study investigates radiomic efficacy in post-surgical traumatic spinal cord injury (SCI), overcoming MRI limitations from metal artifacts to enhance diagnosis, severity assessment, and lesion characterization or prognosis and therapy guidance. Traumatic spinal cord injury (SCI) causes severe neurological deficits. While MRI allows qualitative injury evaluation, standard imaging alone has limitations for precise SCI diagnosis, severity stratification, and pathology characterization, which are needed to guide prognosis and therapy. Radiomics enables quantitative tissue phenotyping by extracting a high-dimensional set of descriptive texture features from medical images. However, the efficacy of postoperative radiomic quantification in the presence of metal-induced MRI artifacts from spinal instrumentation has yet to be fully explored. A total of 50 healthy controls and 12 SCI patients post-stabilization surgery underwent 3D multi-spectral MRI. Automated spinal cord segmentation was followed by radiomic feature extraction. Supervised machine learning categorized SCI versus controls, injury severity, and lesion location relative to instrumentation. Radiomics differentiated SCI patients (Matthews correlation coefficient (MCC) 0.97; accuracy 1.0), categorized injury severity (MCC: 0.95; ACC: 0.98), and localized lesions (MCC: 0.85; ACC: 0.90). Combined T_1_ and T_2_ features outperformed individual modalities across tasks with gradient boosting models showing the highest efficacy. The radiomic framework achieved excellent performance, differentiating SCI from controls and accurately categorizing injury severity. The ability to reliably quantify SCI severity and localization could potentially inform diagnosis, prognosis, and guide therapy. Further research is warranted to validate radiomic SCI biomarkers and explore clinical integration.

## 1. Introduction

Traumatic spinal cord injury (SCI) is a devastating condition affecting millions of individuals worldwide. SCI profoundly impacts physical, psychological, and socioeconomic well-being [1]. In the United States alone, approximately 17,800 new SCI cases occur annually [2]. SCI can damage axons, neurons, glia, and blood vessels, resulting in temporary or permanent sensory and motor deficits below the lesion level [3]. Most SCIs occur at cervical levels, with common causes being motor vehicle collisions, falls, violence, and sports activities [2].

Acute SCI can present with varying clinical manifestations depending on injury level and severity. These include tetraplegia or paraplegia, sensory deficits, autonomic dysfunction affecting cardiovascular, respiratory, and bowel/bladder function, and neuropathic pain. The severity and extent of these manifestations significantly influence patient outcomes and rehabilitation strategies [4].

Magnetic resonance imaging (MRI) is the preferred modality for visualizing the spinal cord and soft tissues [5]. Conventional MRI protocols enable the detection of cord compression, signal changes, edema, hemorrhage, and morphologic alterations after injury. However, qualitative image evaluation has limitations in providing microstructural and functional details needed to guide SCI prognosis and management [6].

Recent advances in quantitative MRI techniques have shown promise in characterizing SCI pathology. Diffusion tensor imaging has demonstrated utility in assessing white matter integrity and predicting functional outcomes [7,8]. Magnetization transfer imaging provides insights into myelin content [7,9], and functional MRI has revealed changes in brain activity following spinal cord injury [10]. While these methods can extract precise biomarkers of post-traumatic cord integrity and function, they each probe specific physiological phenomena in isolation.

Machine learning methods are increasingly being applied to spinal cord imaging analysis. Deep learning approaches have improved segmentation accuracy [11] and pathologic finding detection [12]. Radiomics offers a more holistic approach by extracting multiple descriptive features from medical images through high-throughput data characterization [13]. These methods have shown promise for prognosis in oncology [14]. Recently, radiomic techniques have been explored in spinal cord studies, with Okimatsu et al. developing a model using T2*-weighted MRI to predict neurological outcomes after acute cervical SCI [15]. However, a key challenge is that SCI frequently requires surgical stabilization involving metallic instrumentation. This hardware produces artifacts on conventional postoperative MRI [16] that disrupt quantitative radiomic analyses.

This study aimed to implement a radiomic modeling approach to analyze MRI of the instrumented spinal cord in SCI subjects. Multi-spectral imaging sequences were leveraged to suppress metal artifacts and enable unobstructed radiomic feature extraction at instrumented levels [17]. We hypothesized that radiomic signatures could reliably categorize SCI severity and lesion location. Successfully quantifying MRI traits in instrumented cords could ultimately enable monitoring of traumatic changes to inform SCI diagnosis and therapeutic regimens.

## 2. Materials and Methods

Reporting and analysis in this study followed the CheckList for EvaluAtion of Radiomics research (CLEAR) documentation standard focusing on repeatability, reproducibility, and transparency of radiomic studies [18].

### 2.1. Study Cohorts

This study involved 12 subjects with traumatic SCI who underwent MRI scans at 1-14 months (mean 7.08 ± 4.03) following surgical stabilization at cervical levels using metallic instrumentation. SCI severity was graded using the American Spinal Injury Association (ASIA) Impairment Scale (AIS). The study also included 50 healthy controls with no SCI or cord disorder history. Informed consent was obtained from all participants per our Institutional Review Board protocol. Table 1 summarizes the cohort demographics.

Imaging was performed at 3T (GE Signa Premier) using a 21-channel neurovascular coil. Multi-spectral 3D fast spin echo MRI was acquired to suppress metal artifacts [17]. Isotropic 1.2 mm resolution T_1_- and T_2_-weighted volumes were obtained with 8 spectral bins. Imaging parameters were as follows: TR\TE for T_1_—750/8 ms; for T_2_—2100/60 ms; and ARC 2 × 2 acceleration.

### 2.2. Image Analysis

**Figure 1:**
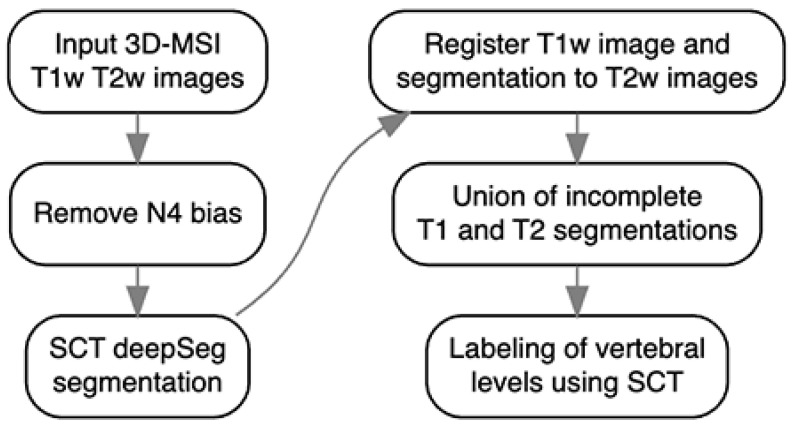
The flowchart depicts the pipeline for segmenting the spinal cord as suggested in [19].

The spinal cord was automatically segmented using a multi-step pipeline (Figure 1) as described in [19]. First, N4 bias field correction was applied to remove image shading artifacts. The Spinal Cord Toolbox (SCT) deep learning model [20] then performed initial segmentation independently on both T_1_ and T_2_ volumes. However, these initial segmentations exhibited intermittent failures near metallic instrumentation where image artifacts were present. To address these failures, the T_1_ data were registered to the T_2_ space using SCT’s registration module. The segmentations were then integrated using a radial basis function (RBF) algorithm, which creates a smooth interpolation between the T_1_ and T_2_ segmentations. The RBF approach was chosen for its robustness to local failures; when one modality’s segmentation fails due to metal artifacts, the algorithm appropriately weights the more reliable segmentation from the other modality in that region. This integrated segmentation leverages complementary information from both imaging sequences to produce a more robust final result. The improved segmentation was then used by SCT to automatically label the vertebral levels [19].

Radiomic feature extraction was performed within cord segmentation using PyRadiomics [21]. A total of 1374 features describing intensity, shape, and texture patterns were generated from original and filtered images, including wavelet, square, square root, logarithm, exponential, gradient, and local binary patterns.

### 2.3. Classification Framework

Three classification tasks were the following: (1) differentiating SCI cases from healthy controls, (2) SCI severity (severe AIS A-B vs. non-severe AIS C-D), and (3) lesion zone (above, at, or below instrumentation level). For each target, models were trained using T_1_, T_2_, or combined T_1_ + T_2_ radiomic features to compare performance. Evaluation metrics were accuracy, the Matthews correlation coefficient (MCC), the F1 score, and the area under the ROC (receiver operating characteristic) curve (AUC).

The radiomic feature sets were input into a supervised machine learning pipeline to differentiate SCI subjects from controls and categorize injury severity and cord location relative to the injury site. The dataset was divided into training (70%), validation (15%), and testing (15%) subsets. An automated modeling framework (H2O AutoML [21]) evaluated various classifiers (random forest, XGBoost, neural networks, etc.) using 5-fold cross-validation on the training data. To address class imbalance in the dataset, oversampling of minority classes was applied during the training process, ensuring more equitable representation and mitigating bias toward the majority class. This approach improves the generalization and reliability of the models, especially for minority class predictions.

Feature selection was automatically performed within the H2O AutoML framework, which identified GBM as the optimal model. The framework leveraged GBM’s inherent feature importance calculation, where features were weighted based on their contribution to decision tree splits and the associated reduction in squared error across the ensemble. Feature importance scores were normalized to a [0, 1] range, enabling systematic identification of the most influential radiomic markers while automatically suppressing less informative features during model optimization. This automated feature selection streamlined the modeling process while maintaining robust predictive performance [22,23].

## 3. Results

**Figure 2:**
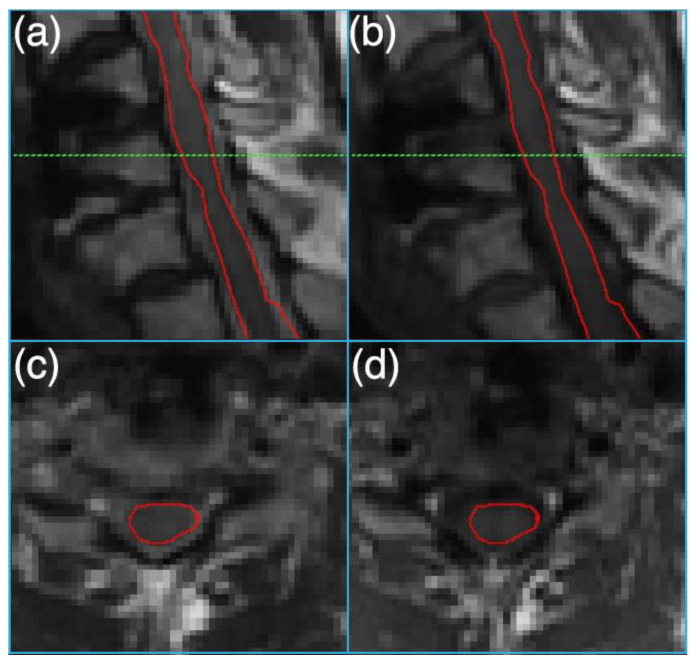
Sagittal (**a**) T_2_-weighted and (**b**) T_1_-weighted 3D-MSI MRI images of an instrumented damaged spinal cord. Axial sections, reformatted at the level of the dashed green line from (**a**,**b**), are shown in (**c**,**d**), respectively. The spinal cord is outlined in red in all images.

**Figure 3:**
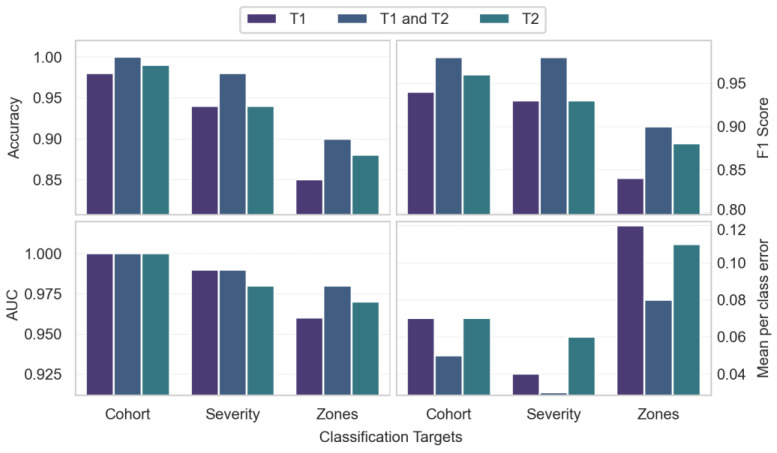
Comparison of accuracy, F1 score, area under the curve (AUC-ROC), and mean per-class error across radiomic classification tasks using T_1_, T_2_, and combined T_1_/T_2_ feature sets. The tasks include categorizing cohorts into healthy or spinal cord injury (SCI) groups, determining injury severity levels, and distinguishing between cord zones relative to the injury site.

Figure 2 depicts sample T_1_- and T_2_-weighted MRI images and the segmented cord on an instrumented slice. As shown in Figure 3, a combined T_1_ and T_2_ feature set achieved strong performance in discriminating between healthy controls and SCI patients, with 0.97 MCC, 0.98 F1 score, 1.00 accuracy, and 1.00 AUC. For predicting injury severity, the T_1_ + T_2_ model again achieved robust performance with 0.95 MCC, 0.98 F1 score, 0.98 accuracy, and 0.99 AUC. The T_2_ model achieved 0.86 MCC and 0.94 accuracy. For lesion zone classification, the T_1_ + T_2_ model performed best with 0.85 MCC, 0.90 F1 score, 0.90 accuracy, and 0.98 AUC. The T_2_ model achieved 0.81 MCC and 0.88 accuracy.

Gradient boosting machine (GBM) models achieved the top performance for most tasks. The only exception was the zone classification task using T_1_ features, for which XGBoost was optimal.

Overall, the combined T_1_ + T_2_ models outperformed individual modalities across tasks. The models demonstrated excellent discrimination for SCI vs. controls and good predictive performance for injury severity. The results were strong but comparatively lower for the more challenging three-class zone classification task.

## 4. Discussion

This preliminary study demonstrates the potential of a radiomic modeling approach for instrumented spinal cord MRI analysis in traumatic SCI. A key advance was the use of multi-spectral imaging to suppress instrumentation artifacts that can distort quantitative feature extraction. The study’s reproducibility is strengthened by the implementation of standardized tools and protocols, including multi-spectral imaging optimized for metal artifact suppression [17], validated automated spinal cord segmentation [19], and standardized radiomic feature extraction following established guidelines [18]. Radiomic SCI characterization could offer advantages over both qualitative evaluation alone and standard diffusion/functional MRI methods that assess specific microstructural or physiological properties in isolation. The high throughput radiomic feature set provides a more comprehensive phenotypic profiling of overall cord tissue traits linked to injury.

The radiomic framework reliably differentiated severe and non-severe SCI categories, achieving robust classification performance. This ability to determine injury severity, which has significant implications for prognosis and therapy, demonstrates clinical utility.

While global accuracy metrics were relatively high across tasks, lower MCC and F1 scores imply that some degree of inter-class imbalance likely exists in the dataset. This imbalance means majority classes were more successfully predicted than minority classes. Techniques such as oversampling of the minority classes or cost-sensitive learning could address this and improve MCC and F1 metrics. Additionally, discrimination power was weaker for more nuanced tasks like severity level or subtle zone differences. These findings warrant focused efforts on feature engineering and model tuning targeting MCC and F1 improvements.

When assessing the advantages of combined T_1_ and T_2_ features versus prolonged scan times, the MCC is particularly informative in the presence of class imbalance. For cohort differentiation, the MCC increase from 0.92 to 0.97 with combined features is substantial. However, the 0.92 baseline already indicates robust predictive power. In efficiency-focused clinical settings, marginal T_1_ + T_2_ benefits may not outweigh longer scans, especially for resource optimization.

For severity classification, the MCC rose slightly from 0.86 to 0.95 with combined features. Although showing an increase, the 0.86 T_2_ baseline is respectable. The slight absolute MCC increase may have limited utility depending on clinical use. T_2_ could suffice when efficiency is critical and acceptable severity discrimination is achievable. However, for applications where severity subtleties carry high stakes, the T_1_ + T_2_ approach may provide value despite a longer scan time.

For multiclass zone classification, the more substantial MCC boost from 0.81 to 0.85 with T_1_ + T_2_ features could justify extra scan time. While context-dependent, this degree of performance lift may warrant dual-acquisition protocols.

The ability to quantitatively characterize post-surgical cord changes through radiomics could provide valuable prognostic information. By reliably categorizing injury severity and location, this approach could help predict functional outcomes, guide rehabilitation strategies, and monitor treatment response. Future longitudinal studies could establish relationships between radiomic features and functional recovery patterns, potentially enabling more personalized treatment planning.

A limitation of this study is the small cohort size, which may impact the generalizability of the results. Future studies with larger and more diverse patient cohorts are warranted to validate the findings and further enhance model robustness and accuracy across different populations. Moreover, although the multi-spectral imaging technique used in this study effectively reduces metallic artifacts, it is important to acknowledge its limitations, particularly in cases with complex or extensive metallic instrumentation. Residual artifacts, while minimized, can still interfere with image quality and may impact the accuracy of radiomic feature extraction. This is especially relevant for texture-based features, which are sensitive to subtle intensity and spatial variations. Such artifacts could introduce variability or bias in the radiomic data, potentially affecting the reliability of the extracted features and subsequent model predictions. While the results of this study demonstrate the potential of radiomic analysis in postoperative spinal cord injury assessment, the influence of residual artifacts warrants further investigation. Future work could explore advanced artifact reduction techniques or post-processing methods to enhance radiomic feature robustness, particularly in challenging cases. Additionally, incorporating quality assurance metrics to quantify artifact levels could help identify and mitigate their impact during the analysis pipeline.

In summary, T_1_ + T_2_ improved performance metrics across tasks. However, clinical value versus efficiency tradeoffs depend on the classification specifics and performance requirements. Further feature engineering or integrating other imaging modalities could refine model performance. More extensive longitudinal studies are essential to fully explore clinical utility. Overall, radiomic modeling shows promise for quantitative SCI MRI, potentially guiding diagnosis and management.

## Figures and Tables

**Table 1:** Characteristics of the study cohorts.

Cohorts	Gender	Count	Age	BMI	ASIA: A	ASIA: B	ASIA: C	ASIA: D
Healthy	Female	25	47.52 ± 15.23	27.22 ± 7.18	-	-	-	-
	Male	25	48.50 ± 16.92	27.84 ± 4.67	-	-	-	-
	Total	50	48.02 ± 15.96	27.54 ± 5.98	-	-	-	-
SCI	Female	6	59.50 ± 18.62	25.57 ± 5.90	0	1	2	3
	Male	6	48.50 ± 21.95	23.52 ± 2.68	2	1	1	2
	Total	12	54.00 ± 20.24	24.54 ± 4.50	2	2	3	5

## Data Availability

The data presented in this study are openly available in Mendeley Data at https://data.mendeley.com/datasets/sjpx7md7cf/1 (accessed on 25 October 2024).
